# Analysis of risk factors and establishment of a predictive model for liver disease severity in metabolic dysfunction–associated fatty liver disease

**DOI:** 10.3389/fmed.2026.1806275

**Published:** 2026-04-20

**Authors:** Ziyu Zhang, Yaqin Zhang, Xinxin Li, Weihua Cao, Wen Deng, Shiyu Wang, Xin Wei, Linmei Yao, Zixuan Gao, Shuojie Wang, Hongxiao Hao, Yuanjiao Gao, Xiaoxue Chen, Yao Xie, Minghui Li

**Affiliations:** 1 Department of Hepatology Division, Beijing Ditan Hospital, Capital Medical University, Beijing, China; 2 Department of Hepatology Division, Peking University Ditan Teaching Hospital, Beijing, China; 3 HBV Infection, Clinical Cure and Immunology Joint Laboratory for Clinical Medicine, Capital Medical University, Beijing, China

**Keywords:** metabolic dysfunction–associated fatty liver disease, fibrosis, inflammation, steatosis, ALT

## Abstract

**Objective:**

To identify risk factors associated with liver disease severity in patients with metabolic dysfunction–associated fatty liver disease (MAFLD) and develop a risk prediction model.

**Methods:**

Clinical data were collected from MAFLD patients diagnosed via liver biopsy at the Second Department of Hepatology, Beijing Ditan Hospital, Capital Medical University, from January 2018 to December 2022. Patients were initially grouped by ALT levels to analyze its limitations in MAFLD diagnosis. Pathological reports were used to classify patients into non-significant (S/F/G < 2) and significant (S/F/G ≥ 2) groups based on fibrosis (S), inflammation (G), and steatosis (F) severity. Logistic regression identified independent risk factors for liver disease severity, and a predictive model was established. Model performance was validated using the area under the curve (AUC).

**Results:**

Significant differences were observed between ALT-normal and ALT-abnormal groups in AST, GGT, ALP, BMI, hyperlipidemia prevalence, age, and diabetes prevalence (*p* < 0.05). Univariate analysis revealed age, BMI, AST, ALB, ALP, TG, and high-calorie diet as significant variables (*p* < 0.05) across inflammation, steatosis, and fibrosis subgroups. When stratified by inflammation severity, multivariate analysis identified sex, age, BMI, ALB, and PcIII as independent predictors of significant inflammation in MAFLD. High BMI and hyperlipidemia were risk factors for MAFLD steatosis. When grouped by fibrosis severity, age, height, BMI, ALB, and PcIII emerged as independent predictors of pathologically significant fibrosis in MAFLD. Patients with G ≥ 2 and/or S ≥ 2 were classified as the significant pathology group, while others formed the non-significant group. A logistic regression model was constructed using regression coefficients and constants, *p* = 1/(1 + e^-Y^), Y = −12.486 + 0.913 × Sex +0.442 × BMI + 0.04 × PcIII-0.085 × ALB. The area under the ROC curve was 0.833 (95% CI: 0.797–0.869), with a maximum Youden index of 0.456, corresponding to sensitivity and specificity of 67.3 and 83.3%, respectively.

**Conclusion:**

Sex, BMI, PcIII, and ALB are closely associated with MAFLD severity. The logistic regression model demonstrates potential clinical utility for predicting significant liver pathology.

## 1 Introduction

Metabolic dysfunction–associated fatty liver disease (MAFLD) is a liver condition that occurs after excluding well-established etiologies such as excessive alcohol consumption (1). As one of the most common drivers of liver diseases, its global prevalence continues to rise (2). Based on distinct histological features, MAFLD can be classified into non-alcoholic fatty liver (NAFL) and non-alcoholic steatohepatitis (NASH). NAFL is characterized by hepatic steatosis with or without mild lobular inflammation, while NASH additionally involves hepatocellular injury (hepatocyte ballooning, diffuse lobular inflammation, and fibrosis) (3). Currently, no approved pharmacotherapy exists for MAFLD, and lifestyle interventions (low-calorie diet and exercise) remain pivotal for weight reduction and disease management (4). Although early-stage isolated steatosis is considered “benign,” its association with fibrosis progression may lead to cirrhosis and hepatocellular carcinoma (HCC) (3). Furthermore, multiple studies indicate cardiovascular disease as a major cause of mortality in MAFLD patients (5). Thus, there is an urgent need for accurate diagnostic methods.

Approximately 25% of the general population is estimated to have MAFLD (6). However, due to the lack of specificity and sensitivity in current diagnostic approaches, the true prevalence remains uncertain (7). Clinical diagnosis predominantly relies on exclusion criteria, and widely used imaging and biochemical tests exhibit inherent limitations (8). Liver biopsy remains the gold standard for differentiating simple steatosis from NASH and staging fibrosis (7). Yet, its invasive nature prevents large-scale application, underscoring the importance of developing novel non-invasive methods to monitor MAFLD progression and treatment response. This study aims to evaluate the predictive performance of combined clinical biomarkers, identify optimal predictors, and provide theoretical insights for assessing liver pathology severity in MAFLD.

## 2 Materials and methods

### 2.1 Study subjects

Patients diagnosed with MAFLD via liver biopsy at the Second Department of Hepatology, Beijing Ditan Hospital, Capital Medical University, from January 2018 to December 2022 were enrolled to investigate clinical factors associated with liver histopathological severity.

### 2.2 Inclusion and exclusion criteria

(1) Exclusion of significant alcohol consumption: Alcohol intake equivalent to ≥30 g ethanol/day (≥210 g/week) for males or ≥20 g/day (≥140 g/week) for females (*Ethanol intake calculation*: Volume (mL) × Alcohol content (%) × 0.8). (2) Exclusion of chronic liver diseases: Viral hepatitis, drug-induced liver injury, autoimmune liver diseases, Wilson’s disease, hepatobiliary infections, and malignancies. (3) Exclusion of medication-induced steatosis: Patients using drugs associated with fatty liver (e.g., glucocorticoids, synthetic estrogens, sodium valproate). (4) Exclusion of systemic disease-related secondary steatosis: Total parenteral nutrition, hypothyroidism, inflammatory bowel disease, etc. (5) Exclusion of incomplete clinical data. (6) Inclusion requirement: Confirmed MAFLD diagnosis via percutaneous liver biopsy.

This study was approved by the Ethics Committee of Beijing Ditan Hospital, which is affiliated with Capital Medical University [Jingdilunke Zi (2018) No. (052–01)]. All patients provided written informed consent before undergoing liver biopsy.

### 2.3 Data collection and clinical parameter assessment

#### 2.3.1 General data

Demographic and clinical data, including sex, age, medical history (hypertension, coronary heart disease, diabetes, hyperlipidemia), personal history, treatment records, and high-calorie dietary habits (defined as a diet rich in fat, sugar, refined carbohydrates, and low in dietary fiber, with daily caloric intake significantly exceeding individual energy requirements), were systematically recorded by trained staff. Height and weight were measured by professionals on the examination day to calculate body mass index [BMI = weight (kg)/height (m)^2^].

#### 2.3.2 Laboratory tests

All participants fasted for >8 h prior to blood collection, venous blood samples were drawn in the morning to assess: Liver function markers: Alanine aminotransferase (ALT), aspartate aminotransferase (AST), gamma-glutamyl transferase (GGT), routine blood tests, fasting blood glucose, triglycerides, high-density lipoprotein (HDL) and low-density lipoprotein (LDL). Clinical biochemical and virological tests were performed at the Clinical Laboratory Center of Beijing Ditan Hospital. Liver function analysis Hitachi 7,180 automated biochemical analyzer (Japan). Hepatocellular carcinoma and cirrhotic ascites evaluation, Ultrasound (Siemens ACUSON X150, United States), Color Doppler flow imaging (Siemens ACUSON X150, United States), Computed tomography (Siemens SOMATOM Definition AS, United States), Magnetic resonance imaging (Siemens MAGNETOM Skyra, United States).

#### 2.3.3 Liver biopsy procedure

All patients provided written informed consent. Patients were positioned in a supine or left lateral decubitus position near the bedside, with the right arm elevated and flexed behind the head. Strict aseptic techniques were maintained by the medical team: operators wore masks, caps and sterile gloves, and the puncture site was disinfected and draped with a sterile fenestrated towel. Local anesthesia was administered using 2% lidocaine for layered infiltration of the skin, muscle, and liver capsule. Under ultrasound guidance, a 16-gage liver biopsy needle was percutaneously inserted during quiet breathing to obtain liver tissue specimens (≥1.5 cm in length). The specimens were fixed, sectioned, and stained with hematoxylin–eosin (HE), Masson’s trichrome, or Sirius red for histological evaluation. All slides were independently reviewed and assessed by experienced pathologists.

#### 2.3.4 Histopathological diagnosis of liver tissue

Liver biopsy specimens were serially sectioned and routinely stained with hematoxylin–eosin (H&E), reticulin, and/or Masson’s trichrome. According to the 2006 NAFLD/NASH diagnostic guidelines issued by the Chinese Society of Hepatology, Chinese Medical Association, histopathological findings were reported as NASH-F (0–4), G (0–3), S (0–4), F: Steatosis grading (0–4), G: Inflammation grading (0–3), S: Fibrosis staging (0–4). For this study, MAFLD patients were stratified into subgroups based on pathological severity: those with scores ≥2 for steatosis (F), inflammation (G), or fibrosis (S) were classified as the significant pathology group.

### 2.4 Statistical analysis

Data were analyzed using IBM SPSS 25. Normally distributed continuous variables are expressed as mean ± standard deviation and compared using *t*-test. Non-normally distributed data are reported as median (interquartile range, IQR) (Q1, Q3) and analyzed with the Mann–Whitney U test. Categorical variables are presented as frequency (percentage, %) and compared via Fisher’s exact test or chi-square test, as appropriate. A *p* < 0.05 indicates statistical significance.

## 3 Results

### 3.1 Demographic and clinical characteristics of patients with normal vs. abnormal ALT levels

#### 3.1.1 Baseline characteristics

A total of 539 eligible patients were included, comprising 57 cases with normal ALT levels and 482 cases with abnormal ALT levels (males: >30 U/L, females: >19 U/L). Among them, 325 were male and 214 females. Patients in the abnormal ALT group were significantly younger and exhibited higher BMI values compared with those in the normal ALT group (*p* < 0.01 and *p* < 0.05, respectively). No significant differences were observed in sex distribution or height between groups. The prevalence of hyperlipidemia was markedly higher, whereas that of diabetes mellitus was lower, among patients with abnormal ALT (both *p* < 0.05). Lifestyle factors, including dietary habits, smoking, alcohol consumption, and hypertension, showed no significant intergroup differences (Table 1).

**Table 1:** Comparison of baseline characteristics between patients with normal and abnormal ALT levels.

Variable	Normal ALT	Abnormal ALT	Z/H	*P*
Sex (male, %)	35(61.4%)	290(60.2%)	0.033	0.857
Age (years)	46.00(36.00, 52.00)	38.00(29.00, 51.00)	−3.099	0.002
Height (m)	1.70(1.62, 1.75)	1.70(1.62, 1.75)	−1.006	0.314
BMI (kg/m^2^)	27.65(24.72, 29.40)	28.48(25.60, 30.06)	−2.419	0.016
Sedentary lifestyle (%)	56(98.2%)	456(94.6%)	–	0.343
High-calorie diet (%)	42(73.7%)	373(77.4%)	0.394	0.530
Smoking (%)	13(22.8%)	86(17.8%)	0.838	0.360
Alcohol consumption (%)	16(28.6%)	120(24.9%)	0.356	0.549
Hypertension (%)	5(8.8%)	67(13.9%)	1.158	0.282
Hyperlipidemia (%)	21(36.8%)	252(52.3%)	4.861	0.027
Diabetes mellitus (%)	12(21.1%)	39(8.1%)	9.996	0.002
Use of lipid-lowering drugs (%)	2(3.5%)	16(3.3%)	–	1.000

#### 3.1.2 Comparison of laboratory parameters between patients with normal and abnormal ALT levels

Significant differences were observed in AST, GGT, and ALP levels between the elevated ALT group and the normal ALT group (p < 0.05), with all three markers significantly higher in the abnormal ALT group. However, no statistically significant differences were found in other laboratory indicators, including HbA1c, insulin, and C-peptide levels (Table 2).

**Table 2:** Comparison of laboratory parameters between patients with normal and abnormal ALT levels.

Project	Normal ALT	Abnormal ALT	*Z*	*P*
HbA1c (%)	5.35(4.78, 6.30)	5.40(4.80, 6.20)	−0.043	0.966
Insulin levels (mU/L)	11.20(6.49, 18.53)	13.87(6.90, 18.35)	−0.424	0.671
C-peptide (ng/mL)	3.60(3.10, 4.80)	3.66(2.90, 4.80)	−0.258	0.797
AST (U/L)	18.50(15.75, 25.00)	51.00(33.00, 81.00)	−10.013	0.000
Total bilirubin (μmol/L)	12.50(10.00, 18.50)	13.00(9.70, 17.00)	−0.639	0.523
Direct bilirubin (μmol/L)	3.75(3.18, 5.80)	4.20(3.00, 5.70)	−1.061	0.289
Albumin (g/L)	45.00(42.00, 49.00)	46.00(43.00, 48.00)	−1.787	0.074
GGT (U/L)	39.50(16.75, 75.50)	71.00(43.00, 121.00)	0.000	0.000
Alkaline phosphatase	73.50(63.50, 89.25)	79.00(65.00, 103.00)	−1.961	0.050
Uric acid (μmol/L)	366.50(317.60, 427.00)	369.00(292.00, 451.00)	−0.314	0.754
Cholesterol (mmol/L)	4.80(3.88, 5.67)	4.75(4.21, 5.59)	−0.186	0.853
Triglycerides (mmol/L)	1.90(1.42, 2.84)	1.70(1.23, 2.49)	−1.889	0.059
HDL (mmol/L)	1.05(0.86, 1.19)	1.04(0.88, 1.21)	−0.139	0.890
LDL (mmol/L)	2.58(1.94, 3.17)	2.84(2.30, 3.39)	−1.919	0.055
Homocysteine (μmol/L)	12.92(9.73, 17.20)	12.00(9.00, 16.00)	−0.615	0.539
AFP (ng/mL)	2.60(2.10, 5.45)	3.20(2.30, 5.30)	−1.061	0.289
Type III procollagen (μg/L)	15.48(12.08, 32.49)	17.40(12.43, 34.56)	−0.161	0.872

The laboratory indicator data above are all non-normally distributed quantitative data, expressed as M (Q1, Q3).

### 3.2 Demographic and clinical characteristics stratified by liver steatosis, inflammation grading, and fibrosis staging in MAFLD

Patients were grouped into non-significant (scores <2) and significant (scores ≥2) pathology groups based on histopathological severity of fibrosis (S), inflammation (G), and steatosis (F). General characteristics, lifestyle factors, and laboratory parameters were compared between groups using the Mann–Whitney U test (Table 3).

**Table 3:** Comparative analysis of demographic and clinical features across NAFLD severity subgroups.

Project	G<2	G ≥ 2	*Z*	S<2	S ≥ 2	*Z*	F<2	F ≥ 2	*Z*
Age (years)	36.00(28.00, 48.50)	46.00(34.00, 55.00)	−4.243^**^	37.00(28.00, 49.00)	46.50(37.00, 55.00)	−4.382^**^	45.00(34.00, 54.00)	36.00(27.00, 47.00)	−5.721^**^
Height (m)	1.70(1.64, 1.76)	1.65(1.59, 1.74)	−4.997^**^	1.71(1.64, 1.76)	1.63(1.59, 1.72)	−5.582^**^	1.69(1.61, 1.75)	1.70(1.62, 1.75)	−0.517
BMI (kg/m^2^)	27.89(24.92, 29.66)	29.41(28.08, 30.58)	−7.682^**^	27.90(24.96, 29.59)	29.72(28.54, 30.75)	−7.413^**^	25.96(24.69, 29.15)	29.06(27.43, 30.10)	−7.829^**^
HbA1c (%)	5.30(4.80, 6.00)	5.60(4.98, 6.60)	−2.918^**^	5.30(4.80, 6.00)	5.60(5.10, 6.60)	−3.166^**^	5.40(4.90, 6.10)	5.40(4.80, 6.30)	−0.099
C-peptide (ng/mL)	3.60(2.90, 4.80)	3.90(3.20, 4.80)	−1.630	3.60(2.90, 4.75)	3.92(3.20, 5.30)	−2.428^*^	3.70(2.90, 4.80)	3.60(3.10, 4.80)	−0.173
ALT (U/L)	76.00(42.00, 133.00)	112.00(63.00, 161.00)	−4.569^**^	84.70(47.40, 147.50)	83.50(47.00, 145.25)	−0.210	58.50(30.75, 105.50)	104.00(62.00, 158.00)	−6.486^**^
AST (U/L)	39.00(27.00, 61.00)	71.00(44.00, 110.00)	−8.646^**^	43.00(29.00, 72.00)	58.50(34.25, 94.75)	−3.556^**^	34.00(24.00, 60.55)	53.00(35.00, 83.00)	−5.249^**^
Total bilirubin (μmol/L)	13.00(10.00, 17.00)	13.00(9.60, 17.00)	−1.174	12.05(9.90, 17.00)	13.00(9.65, 17.00)	−0.203	12.00(10.00, 17.00)	13.00(9.60, 17.00)	−0.270
Direct bilirubin (μmol/L)	4.00(3.00, 5.90)	4.40(3.10, 5.60)	−0.697	4.00(3.00, 5.68)	4.60(3.23, 5.80)	−1.975^*^	4.50(3.00, 6.20)	4.00(3.00, 5.60)	−0.973
Albumin (g/L)	47.00(44.00, 49.00)	45.00(41.00, 47.00)	−4.468^**^	47.00(44.00, 49.00)	45.00(42.00, 47.00)	−3.243^**^	45.00(42.00, 48.00)	47.00(44.00, 51.00)	−2.776^**^
GGT (U/L)	61.80(35.00, 111.50)	83.00(52.00, 125.00)	−3.026^**^	63.00(35.00, 114.25)	79.50(51.53, 125.00)	−2.741^**^	68.00(31.00, 131.25)	70.00(43.00, 106.00)	−1.303
Alkaline phosphatase	77.00(64.00, 97.00)	83.00(67.00, 108.00)	−2.621^**^	77.70(64.00, 100.75)	81.50(67.00, 102.50)	−1.444	77.75(65.75, 99.50)	79.00(65.00, 102.00)	−0.106
Cholesterol (mmol/L)	4.83(4.30, 5.62)	4.70(4.04, 5.55)	−0.609	4.85(4.25, 5.62)	4.53(4.05, 5.40)	−1.552	4.68(4.06, 5.39)	4.87(4.28, 5.64)	−2.475^*^
Triglycerides (mmol/L)	1.77(1.29, 2.60)	1.54(1.10, 2.27)	−2.087^*^	1.77(1.27, 2.59)	1.51(1.09, 2.29)	−1.981^*^	1.67(1.15, 2.29)	1.75(1.30, 2.65)	−1.976^*^
HDL (mmol/L)	1.04(0.89, 1.22)	1.04(0.87, 1.16)	−0.883	1.04(0.89, 1.22)	1.03(0.86, 1.15)	−1.133	1.04(0.88, 1.21)	1.04(0.89, 1.21)	−0.039
LDL (mmol/L)	2.84(2.29, 3.33)	2.79(2.15, 3.37)	−0.659	2.86(2.27, 3.42)	2.74(2.17, 3.14)	−1.564	2.72(2.14, 3.19)	2.91(2.30, 3.39)	−2.805^**^
Homocysteine (μmol/L)	12.00(8.85, 15.75)	12.50(9.34, 17.00)	−0.880	12.00(8.87, 15.98)	12.90(9.37, 16.93)	−0.660	12.00(9.25, 15.82)	12.22(8.84, 16.67)	−0.129
AFP (ng/mL)	3.10(2.20, 4.90)	3.60(2.50, 6.40)	−2.321^*^	3.10(2.20, 4.80)	3.95(2.50, 7.08)	−2.608^**^	3.25(2.20, 5.30)	3.10(2.30, 5.50)	−0.329
Type III procollagen (μg/L)	16.20(11.83, 25.36)	21.25(12.91, 46.33)	−4.813^**^	15.99(11.99, 26.07)	32.08(13.12, 51.46)	−5.470^**^	16.57(10.31, 35.26)	17.40(12.50, 33.66)	−0.893

Project	G<2	G ≥ 2	H	S<2	S ≥ 2	H	F<2	F ≥ 2	H
Sex (male, %)	251(68.2%)	74(43.3%)	30.314^**^	268(64.6%)	57(46.0%)	13.813^**^	126(58.3%)	199(61.6%)	0.580
Sedentary lifestyle (%)	350(95.1%)	162(94.7%)	0.034	393(94.7%)	119(96.0%)	0.323	205(94.9%)	307(95.0%)	0.005
High-calorie diet (%)	268(72.8%)	147(86.0%)	11.378^**^	311(74.9%)	104(83.9%)	4.299^*^	138(63.9%)	277(85.8%)	34.951^**^
Smoking (%)	74(20.1%)	25(14.6%)	2.346	76(18.3%)	23(18.5%)	0.004	47(21.8%)	52(16.1%)	2.766
Alcohol consumption (%)	99(26.9%)	37(21.6%)	1.715	101(24.3%)	35(28.2%)	0.765	61(28.2%)	75(23.2%)	1.730
患Hypertension (%)	43(11.7%)	29(17.0%)	2.806^*^	51(12.3%)	21(16.9%)	1.781	28(13.0%)	44(13.6%)	0.049
Hyperlipidemia (%)	189(51.4%)	84(49.1%)	0.234	213(51.3%)	60(48.4%)	0.330	92(42.6%)	181(56.0%)	9.360 ^**^
Diabetes mellitus (%)	27(7.3%)	24(14.0%)	6.114^*^	31(7.5%)	20(16.1%)	8.356^**^	22(10.2%)	29(9.0%)	0.220
Use of lipid-lowering drugs (%)	14(3.8%)	4(2.3%)	0.776	15(3.6%)	3(2.4%)	–	7(3.2%)	11(3.4%)	0.011

^*^P < 0.05, ^**^P < 0.01.

#### 3.2.1 Inflammation severity (G ≥ 2 vs. G < 2)

Compared with the non-significant group, patients with significant inflammation had higher levels of alpha-fetoprotein (AFP), age, BMI, HbA1c, ALT, AST, GGT, ALP, and procollagen III (PCIII), as well as a higher prevalence of high-calorie dietary habits and diabetes mellitus (all *p* < 0.05). In contrast, triglycerides (TG), albumin (ALB), and the proportion of male patients were significantly lower in the significant inflammation group (*p* < 0.05).

#### 3.2.2 Fibrosis severity (S ≥ 2 vs. S < 2)

The significant fibrosis group showed higher values of direct bilirubin (Dbil), C-peptide, HbA1c, AST, GGT, AFP, PCIII, age, height, and BMI, along with a higher prevalence of high-calorie diet and diabetes mellitus (*p* < 0.05). Conversely, TG, ALB, and the proportion of male patients were lower in the significant fibrosis group (p < 0.05).

#### 3.2.3 Steatosis severity (*F* ≥ 2 vs. *F* < 2)

Patients with significant steatosis exhibited higher levels of total cholesterol (TC), TG, BMI, AST, ALT, ALB, and LDL, and a greater prevalence of high-calorie dietary habits and hyperlipidemia compared with those in the non-significant group (*p* < 0.05).

#### 3.2.4 Overall trends

Across all subgroup analyses based on inflammation, fibrosis, and steatosis severity, consistent intergroup differences were observed in age, BMI, AST, ALB, ALP, TG, and the prevalence of high-calorie dietary habits (*p* < 0.05).

### 3.3 Analysis of factors associated with MAFLD inflammation severity, fibrosis staging, and steatosis degree

#### 3.3.1 Analysis of factors associated with MAFLD inflammation severity

Mild-to-moderate and severe inflammation grades in MAFLD were analyzed, with inflammation severity as the dependent variable. Univariate regression identified sex, age, BMI, HbA1c, ALT, AST, ALB, PCIII, high-calorie diet, and diabetes as significant correlates of inflammation severity. Subsequent multivariate regression confirmed sex (male), age, BMI, PCIII as risk factors and ALB as a protective factor (Table 4). Logistic Predictive Model for Significant Inflammation: *p* = 1/(1 + e^-Y^), Y = −11.392 + 0.846 × sex+0.026 × age+0.393 × BMI + 0.031 × PcIII-0.081 × ALB. Age, BMI, PCIII, and ALB values were entered as continuous variables.

**Table 4:** Univariate and multivariate analyses of hepatic inflammation severity, liver fibrosis stage, and hepatic steatosis degree.

Project	*G*				*F*				*S*			
	OR (U)	95%CI (U)	OR (M)	95%CI (M)	OR (U)	95%CI (U)	OR (M)	95%CI (M)	OR (U)	95%CI (U)	OR (M)	95%CI (M)
Age	1.031	1.016~1.046	1.026^*^	1.004~1.050	0.959	0.946~0.973	0.970^**^	0.953~0.986	1.036	1.019~1.053	1.027 ^*^	1.003~1.053
Height	1.003	0.987~1.021	–	–	1.952	0.250~15.249			0.001	0.000 ~0.008	0.001 ^**^	0.000~0.091
BMI	1.349	1.242~1.465	1.481^**^	1.311~1.673	1.33	1.237~1.431	1.263^**^	1.159~1.375	1.375	1.252~1.511	1.527 ^**^	1.326~1.758
HbA1c	1.259	1.076~1.473	1.117	0.894~1.397	1.034	0.887~1.205	–	–	1.270	1.074~1.502	1.073	0.844~1.364
C-peptide	1.02	0.951~1.093	–	–	1.006	0.940~1.077	–	–	1.041	0.967~1.119	–	–
ALT	1.002	1.001~1.004	1.001	0.998~1.004	1.004	1.002~1.006	1.002	1.000 ~1.005	1.000	0.999~1.002	–	–
AST	1.008	1.005~1.012	1.004	0.998~1.009	1.004	1.000~1.007	1.000	0.996~1.005	1.003	1.000 ~1.005	1.000	0.997~1.004
Tbil	0.991	0.973~1.009	–	–	0.997	0.983~1.013	–	–	0.981	0.958~1.005	–	–
Dbil	1.006	0.987~1.026	–	–	0.987	0.968~1.007	–	–	0.996	0.972~1.021	–	–
ALB	0.917	0.877~0.958	0.922^**^	0.865~0.983	1.074	1.030~1.120	1.018	0.968~1.072	0.930	0.887~0.975	0.898 ^**^	0.837~0.963
GGT	1.000	0.999~1.001	–	–	0.999	0.998~1.000	–	–	1.000	0.999~1.002	–	–
ALP	1.000	0.999~1.002	–	–	0.999	0.997~1.001	–	–	1.000	0.998~1.002	–	–
TC	0.954	0.801~1.136	–	–	1.211	1.022~1.435	1.119	0.897~1.395	0.866	0.711~1.054	–	–
TG	1.002	0.989~1.016	–	–	1.094	0.981~1.220			1.006	0.992~1.019	–	–
HDL	0.706	0.364~1.371	–	–	1.031	0.849~1.252			0.672	0.319~1.415	–	–
LDL	1.014	0.844~1.219	–	–	1.283	1.047~1.573	1.052	0.836~1.324	0.805	0.635~1.020	–	–
HCY	1.011	0.991~1.033	–	–	1.02	0.998~1.043	–	–	1.006	0.983~1.029	–	–
AFP	1.003	0.989~1.017	–	–	0.983	0.949~1.019		–	1.005	0.991~1.019	–	–
PcIII,	1.034	1.023~1.046	1.032^**^	1.018~1.046	1.001	0.991~1.012		–	1.041	1.028~1.054	1.041 ^**^	1.026~1.056
^*^P < 0.05, ^**^P < 0.01. U, Univariate Analyses; M, Multivariate Analyses.												

Project	*G*				*F*				*S*			
	OR (U)	95%CI (U)	OR (M)	95%CI (M)	OR (U)	95%CI (U)	OR (M)	95%CI (M)	OR (U)	95%CI (U)	OR (M)	95%CI (M)
Sex (male, %)	2.754^**^	1.893~4.006	2.331^**^	1.311~4.143	0.881	0.62~1.253	–	–	2.079	1.382~3.128	1.578	0.673~3.701
Sedentary lifestyle (%)	0.914	0.402~2.079	–	–	1.023	0.465~2.249	–	–	1.310	0.485~3.535	–	–
High-calorie diet (%)	2.369	1.442~3.890	0.930	0.448~1.932	3.379	2.225~5.131	1.576	0.950~2.615	1.813	1.059~3.103	1.204	0.530~2.735
Smoking (%)	0.690	0.420~1.132	–	–	0.695	0.448~1.078	–	–	1.036	0.618~1.739	–	–
Alcohol consumption (%)	0.759	0.493~1.168	–	–	0.756	0.510~1.122	–	–	1.247	0.793~1.959	–	–
Hypertension (%)	1.566	0.939~2.610	–	–	1.067	0.641~1.774	–	–	1.484	0.853~2.582	–	–
Hyperlipidemia (%)	0.936	0.650~1.347	–	–	1.743	1.230~2.469	1.554^**^	1.035~2.333	0.918	0.613~1.374	–	–
Diabetes mellitus (%)	2.171	1.206~3.908	0.942	0.418~2.123	0.917	0.508~1.655	–	–	2.307	1.252~4.252	1.136	0.489~2.642
Use of lipid-lowering drugs (%)	0.613	0.199~1.891	–	–	1.059	0.404~2.777	–	–	0.672	0.191~2.361	–	–

^*^P < 0.05, ^**^P < 0.01.

**Figure 1:**
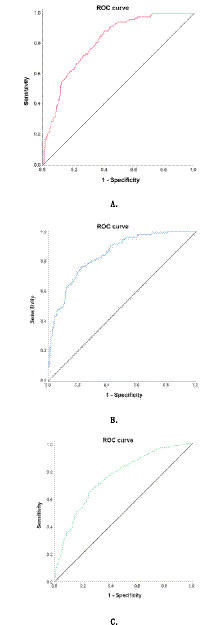
ROC curves of the predictive models for significant inflammation **(A)**, fibrosis **(B)**, and steatosis **(C)** in MAFLD.

The model-generated inflammation risk scores were evaluated via ROC analysis (Figure 1A), achieving a maximum Youden index of 0.238 and an AUC of 0.828 (95% CI: 0.790–0.866), with sensitivity 85.1% and specificity 65.1%.

#### 3.3.2 Analysis of factors associated with MAFLD fibrosis severity

When fibrosis severity was analyzed as the dependent variable, univariate regression identified male sex, age, height, BMI, HbA1c, AST, ALB, LDL, PCIII, high-calorie diet, and diabetes as factors significantly correlated with fibrosis progression. Multivariate regression confirmed age, height, BMI, ALB, and PCIII as independent predictors of histologically significant fibrosis (Table 4). Logistic Predictive Model for Significant Fibrosis: *p* = 1/(1 + e^-Y^), Y = 1.782 + 0.027 × age-7.451 × height+0.423 × BMI + 0.04 × PcIII-0.107 × ALB (Age, height, BMI, PCIII, and ALB values were entered as continuous variables).

The fibrosis risk scores derived from this model were validated by ROC analysis (Figure 1B), demonstrating a maximum Youden index of 0.247 and an AUC of 0.842 (95% CI: 0.802–0.883), with sensitivity 76.4% and specificity 77.3%.

#### 3.3.3 Analysis of factors associated with MAFLD steatosis severity

After incorporating parameters related to histopathological steatosis severity into the analysis (with steatosis degree as the dependent variable), univariate regression identified age, BMI, ALT, AST, ALB, TC, LDL, high-calorie diet, and hyperlipidemia as significant correlates of steatosis severity. Multivariate analysis revealed elevated BMI and hyperlipidemia as risk factors, while age emerged as a protective factor against significant steatosis (Table 4). Logistic Predictive Model for Significant Steatosis: *p* = 1/(1 + e-Y), Y = −7.143–0.031 × age+0.233 × BMI + 0.441 × hyperlipidemia (yes = 1, no = 0), (Age and BMI values were entered as continuous measurements). Steatosis risk scores derived from this model were validated via ROC analysis (Figure 1C), yielding a maximum Youden index of 0.630 and an AUC of 0.759 (95% CI: 0.718–0.801), with sensitivity 66.6% and specificity 74.0%.

### 3.4 Risk factor analysis for significant MAFLD histopathological lesions

Based on the comprehensive findings, patients were categorized into a significant MAFLD group (defined by histopathological scores G ≥ 2 and/or S ≥ 2) and a non-significant MAFLD group. Non-parametric testing of continuous variables between groups (as shown in Table 5) demonstrated that the non-significant group had higher albumin (ALB) levels (*p* < 0.05), whereas the significant group exhibited significantly elevated values in age, BMI, HbA1c, C-peptide, ALT, AST, AFP, GGT, PCIII, and liver stiffness (*p* < 0.05). Furthermore, comparisons of general characteristics and lifestyle habits revealed that the significant group had a higher proportion of males, a greater prevalence of high-calorie dietary habits, and a higher rate of diabetes compared to the non-significant group (*p* < 0.05).

**Table 5:** Comparison between non-significant and significant MAFLD groups.

Project	Non-significant group	Non-significant group	Z/H	*P*
Age (years)	36.00(28.00, 49.00)	45.00(32.00, 54.75)	−4.082	0.000
BMI (kg/m^2^)	27.43(24.84, 29.40)	29.45(28.39, 30.49)	−5.730	0.000
HbA1c (%)	5.30(4.80, 6.00)	5.60(5.10, 6.58)	−8.742	0.000
C-peptide (ng/mL)	3.60(2.82, 4.60)	3.92(3.20, 5.20)	−3.881	0.000
ALT (U/L)	78.00(42.00, 135.00)	97.50(55.50, 158.75)	−3.680	0.000
AST (U/L)	39.00(27.00, 61.00)	64.00(38.25, 101.50)	−7.829	0.000
Total bilirubin (μmol/L)	12.00(10.00, 17.00)	13.00(9.60, 17.00)	−0.376	0.707
Direct bilirubin (μmol/L)	4.00(2.90, 5.90)	4.50(3.13, 5.60)	−1.456	0.145
Albumin (g/L)	47.00(44.00, 49.00)	45.00(42.00, 47.75)	−3.886	0.000
GGT (U/L)	60.00(34.00, 112.00)	79.00(50.78, 124.50)	−3.085	0.002
Alkaline phosphatase	77.00(64.00, 97.00)	82.00(67.00, 104.00)	−1.950	0.051
Cholesterol (mmol/L)	4.81(4.25, 5.62)	4.74(4.08, 5.54)	−0.236	0.813
Triglycerides (mmol/L)	1.75(1.28, 2.59)	1.65(1.16, 2.38)	−1.723	0.085
HDL (mmol/L)	1.04(0.89, 1.22)	1.04(0.87, 1.16)	−0.637	0.524
LDL (mmol/L)	2.83(2.27, 3.35)	2.82(2.24, 3.37)	−0.640	0.522
Homocysteine (μmol/L)	12.00(8.86, 15.69)	12.67(9.33, 17.24)	−1.004	0.316
AFP (ng/mL)	3.10(2.20, 4.90)	3.60(2.43, 5.88)	−2.162	0.031
Type III procollagen (μg/L)	15.86(11.68, 23.60)	22.05(12.99, 46.32)	−5.457	0.000
Sex (male, %)	234(68.6%)	91(45.5%)	28.099	0.000
Sedentary lifestyle (%)	324(95.0%)	190(95.0%)	0.000	0.994
High-calorie diet (%)	245(71.8%)	171(85.5%)	13.225	0.000
Smoking (%)	66(19.4%)	33(16.5%)	0.687	0.407
Alcohol consumption (%)	91(26.7%)	45(22.5%)	1.174	0.279
Hypertension (%)	38(11.1%)	34(17.0%)	3.747	0.053
Hyperlipidemia (%)	172(50.4%)	101(50.5%)	0.000	0.989
Diabetes mellitus (%)	20(5.9%)	31(15.5%)	13.706	0.000
Use of lipid-lowering drugs (%)	13(3.8%)	5(2.5%)	0.675	0.411

Univariate and multivariate logistic regression analyses were performed with MAFLD significance (significant vs. non-significant groups) as the dependent variable. Univariate analysis identified relevant factors, which were subsequently incorporated into multivariate analysis. Results revealed male sex, BMI, and PCIII as independent risk factors for significant MAFLD, while ALB served as a protective factor (Table 6).

**Table 6:** Univariate and multivariate logistic regression analysis for the significant MAFLD group.

Project	P (U)	OR (U)	95%CI (U)	P (M)	OR (M)	95%CI (M)
Age (years)	0.000	1.028	1.014 ~ 1.043	0.085	1.020	0.997 ~ 1.044
BMI (kg/m^2^)	0.000	1.388	1.280 ~ 1.505	0.000	1.556	1.374 ~ 1.762
HbA1c (%)	0.000	1.351	1.156 ~ 1.580	0.241	1.147	0.912 ~ 1.441
C-peptide (ng/mL)	0.359	1.032	0.965 ~ 1.104	–	–	–
ALT (U/L)	0.013	1.002	1.000 ~ 1.003	0.439	0.999	0.995 ~ 1.002
AST (U/L)	0.000	1.008	1.005 ~ 1.012	0.051	1.007	1.000 ~ 1.015
Total bilirubin (umol/L)	0.338	0.992	0.975 ~ 1.009	–	–	–
Direct bilirubin (umol/L)	0.719	1.004	0.984 ~ 1.023	–	–	–
Albumin (g/L)	0.001	0.933	0.894 ~ 0.973	0.009	0.918	0.861 ~ 0.979
GGT (U/L)	0.472	1.000	0.999 ~ 1.001	–	–	–
Alkaline phosphatase	0.887	1.000	0.999 ~ 1.002	–	–	–
Cholesterol (mmol/L)	0.710	0.969	0.819 ~ 1.145	–	–	–
Triglycerides (mmol/L)	0.801	1.002	0.988 ~ 1.015	–	–	–
HDL (mmol/L)	0.397	0.764	0.410 ~ 1.424	–	–	–
LDL (mmol/L)	0.993	1.001	0.838 ~ 1.196	–	–	–
Homocysteine (μmol/L)	0.117	1.016	0.996 ~ 1.037	–	–	–
AFP (ng/mL)	0.825	1.002	0.988 ~ 1.015	–	–	–
Type III procollagen (μg/L)	0.000	1.04	1.028 ~ 1.053	0.000	1.041	1.026 ~ 1.056
LSM (Kpa)	0.000	1.266	1.198 ~ 1.337	–	–	–
Sex (male, %)	0.000	2.646	1.841 ~ 3.802	0.003	2.491	1.378 ~ 4.501
Sedentary lifestyle (%)	0.991	0.996	0.447 ~ 2.219	–	–	–
High-calorie diet (%)	0.000	2.339	1.469 ~ 3.724	0.961	0.982	0.468 ~ 2.059
Smoking (%)	0.411	0.824	0.520 ~ 1.306	–	–	–
Alcohol consumption (%)	0.273	0.795	0.528 ~ 1.198	–	–	–
Hypertension (%)	0.054	1.637	0.992 ~ 2.699	–	–	–
Hyperlipidemia (%)	0.978	1.005	0.708 ~ 1.427	–	–	–
Diabetes mellitus (%)	0.001	2.856	1.574 ~ 5.184	0.462	1.370	0.592 ~ 3.169
Use of lipid-lowering drugs (%)	0.416	0.648	0.227 ~ 1.845	–	–	–

Logistic Predictive Model for MAFLD Significance: *p* = 1/(1 + e^-Y^), Y = −12.486 + 0.913 × Sex (male = 1, female = 0) + 0.442 × BMI + 0.04 × PcIII-0.085 × ALB, (BMI, PCIII, and ALB were entered as continuous variables).

**Figure 2:**
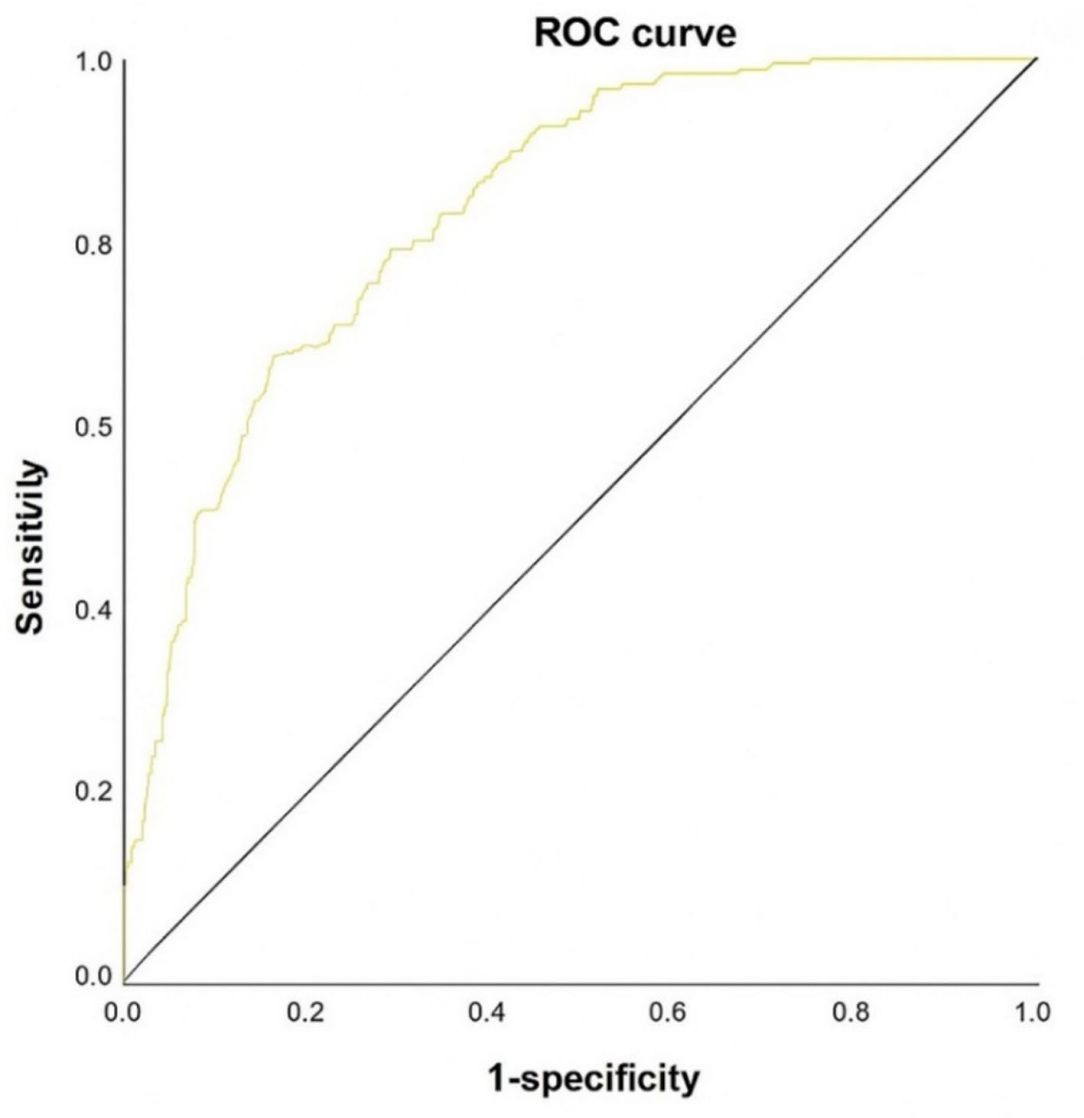
ROC curve of the predictive model for significant MAFLD.

The model-derived MAFLD significance scores were evaluated via ROC analysis, yielding a maximum Youden index of 0.456 and an AUC of 0.833 (95% CI: 0.797–0.869), with sensitivity 67.3% and specificity 83.3% (Figure 2).

## 4 Discussion

This study analyzed 539 patients diagnosed with MAFLD, confirming multiple known clinical risk factors while also establishing non-invasive diagnostic models tailored to distinct histopathological outcomes.

ALT, a sensitive hepatic enzyme reflecting liver inflammation and injury (9), has historically guided clinical focus toward patients with elevated levels. However, prior research (10) reported that 80.3% of suspected MAFLD patients had normal ALT levels, and our study found approximately 10% of MAFLD patients exhibited normal ALT. Although the elevated ALT group showed significantly higher AST, GGT, ALP, BMI, and hyperlipidemia prevalence compared to the normal ALT group (*p* < 0.05), the latter group was younger and had a lower diabetes prevalence (*p* < 0.05). Critically, these findings underscore that normal ALT levels do not eliminate the risk of disease progression, highlighting the diagnostic limitations of relying solely on ALT for MAFLD evaluation.

MAFLD encompasses a continuum of liver lesions, ranging from simple steatosis (≥5% triglyceride accumulation) to non-alcoholic steatohepatitis (NASH) characterized by hepatic lipid deposition and inflammation. Notably, 20% of NASH patients develop hepatic fibrosis, and a subset may progress to hepatocellular carcinoma (HCC) even in the absence of cirrhosis (11). In this study, MAFLD patients were stratified into non-significant (pathology scores <2) and significant groups (scores ≥2) based on fibrosis (S), inflammation (G), and steatosis (F) severity. Across all stratification criteria (inflammatory, steatotic, or fibrotic severity), statistically significant differences (*p* < 0.05) were observed between groups in age, BMI, AST, ALB, ALP, TG levels, and the proportion of individuals with high-calorie dietary habits.

Inflammation is a pivotal driver of MAFLD progression from simple steatosis to advanced hepatic injury. In this study, stratification by inflammatory severity showed that 370 patients (68.09% of the cohort) fell into the non-significant inflammation group, whereas those with significant inflammation exhibited markedly higher levels of AFP, age, BMI, HbA1c, ALT, AST, GGT, ALP, PCIII, and greater proportions of individuals with high-calorie dietary patterns and diabetes (*p* < 0.05). Multivariate logistic regression identified sex, age, BMI, ALB, and PCIII as independent predictors of significant inflammation, suggesting that female sex, younger age, weight reduction, lower PCIII levels, and higher ALB serve as protective factors. Mechanistically, estrogen-mediated enhancement of hepatic metabolic activity in premenopausal women helps restrain inflammatory escalation, whereas postmenopausal metabolic decline is associated with increased susceptibility to MAFLD (12). Obesity further accelerates inflammatory progression through immune-cell infiltration and activation in adipose tissue, which amplifies proinflammatory cytokine release (12). Low serum albumin may reflect systemic inflammation (13), although its predictive value and independent biological role remain uncertain (14). Consistent with these mechanistic insights, ALB appeared protective against hepatic inflammation in this cohort, though this association warrants further validation. Taken together, these findings indicate that when liver histopathology is unavailable, an integrated assessment of demographic and biochemical parameters may offer a clinically practical means of predicting inflammatory severity in MAFLD.

This study identifies elevated BMI and hyperlipidemia as principal risk factors for MAFLD-associated steatosis. Obesity-related disruption of adipokine homeostasis—most notably the reduction in adiponectin—exacerbates hepatic lipid accumulation and potentiates inflammatory and fibrogenic pathways (12). In chronic liver disease (CLD), sustained injury perturbs normal repair mechanisms, driving fibroblast activation into collagen-producing myofibroblasts and leading to excessive extracellular matrix deposition, matrix remodeling, and amplified inflammatory signaling (15, 16). Adipose-derived inflammatory cues further accelerate hepatic stellate-cell activation and fibrosis progression (17). In this study, stratification by fibrosis severity revealed age, height, BMI, ALB, and PCIII as independent predictors of significant MAFLD-related fibrosis. These observations are consistent with epidemiological evidence demonstrating a positive association between BMI and fibrosis risk, now recognized as an independent determinant of fibrotic progression in metabolic dysfunction–associated fatty liver disease (18, 19). Prior studies also indicate a lower risk of fibrosis progression among females (20, 21), a pattern that contrasts with the male-predominant susceptibility observed here. PCIII, one of the most abundant interstitial collagens in fibrotic liver tissue, is a validated biomarker of hepatic fibrogenesis (22) and has shown diagnostic utility even in HIV-associated liver disease, where cross-linked type III collagen formation correlates strongly with fibrosis burden (23). Collectively, the associations between elevated BMI, hyperlipidemia, and steatosis underscore the clinical importance of weight reduction and lipid-lowering strategies in attenuating histopathological progression in MAFLD.

In this cohort, patients were classified into significant (G ≥ 2 and/or S ≥ 2) and non-significant histopathological groups. Compared with those without substantial injury, individuals in the non-significant group exhibited preserved hepatic synthetic function, reflected by higher albumin levels. By contrast, patients with significant pathological changes were characterized by older age, higher BMI, impaired glucose metabolism, elevated hepatocellular injury markers, increased extracellular matrix turnover, greater liver stiffness, and a higher prevalence of high-calorie dietary patterns and diabetes, underscoring the tight link between metabolic derangements and histological severity in MAFLD. Multivariate modeling further identified male sex, BMI, and PCIII as independent determinants of significant disease, whereas albumin emerged as a protective factor. A logistic regression model constructed from routine clinical parameters showed moderate discriminatory performance for predicting histopathological severity (AUC = 0.833; 95% CI, 0.797–0.869). This relatively moderate sensitivity may reflect the heterogeneity of disease severity in biopsy-confirmed MAFLD and the fact that the model was derived from routinely available clinical parameters rather than specialized biomarkers. Although sensitivity and specificity leave room for improvement, the model nonetheless offers clinically accessible stratification that may facilitate earlier intervention and refined prognostication. Collectively, these findings highlight that an integrated assessment of sex, BMI, PCIII, and albumin provides mechanistic and clinically actionable insight into the progression of MAFLD across steatosis, inflammation, and fibrosis.

Although this study was based on a relatively large biopsy-confirmed cohort and multivariable modeling, several limitations should be considered when interpreting the findings. Although the model showed acceptable design may introduce selection bias and residual confounding that cannot be fully excluded. Although the model showed acceptable discrimination within this cohort, it has not been externally validated, and its performance in other populations or clinical settings therefore remains uncertain. In addition, the model demonstrated moderate sensitivity, which may limit its ability to identify all cases of severe disease, suggesting that it should be interpreted as a risk stratification tool rather than a replacement for clinical evaluation. We also did not directly compare the model with commonly used noninvasive fibrosis scores such as FIB-4 or APRI, which limits the ability to place its performance in the context of existing tools used in routine practice. Because all participants underwent liver biopsy, the study population may represent a selected subgroup of patients rather than the broader population encountered in everyday clinical care.

## Data Availability

The raw data supporting the conclusions of this article will be made available by the authors, without undue reservation.
